# Regression-based approach for testing the association between multi-region haplotype configuration and complex trait

**DOI:** 10.1186/1471-2156-10-56

**Published:** 2009-09-17

**Authors:** Yanling Hu, Sinnwell Jason, Qishan Wang, Yuchun Pan, Xiangzhe Zhang, Hongbo Zhao, Changlong Li, Libin Sun

**Affiliations:** 1 School of Agriculture and Biology, Shanghai Jiao Tong University, Shanghai, PR China; 2 Mayo Clinic, Division of Biomedical Statistics and Informatics, Rochester, Minnesota, USA; 3 Zhejiang Provincial Laboratory of Experimental Animals & Non-clinical Studies, Hangzhou, PR China; 4 Shanghai Institute of Veterinary Hygiene, Shanghai, PR China

## Abstract

**Background:**

It is quite common that the genetic architecture of complex traits involves many genes and their interactions. Therefore, dealing with multiple unlinked genomic regions simultaneously is desirable.

**Results:**

In this paper we develop a regression-based approach to assess the interactions of haplotypes that belong to different unlinked regions, and we use score statistics to test the null hypothesis of non-genetic association. Additionally, multiple marker combinations at each unlinked region are considered. The multiple tests are settled via the *minP *approach. The *P *value of the "best" multi-region multi-marker configuration is corrected via Monte-Carlo simulations. Through simulation studies, we assess the performance of the proposed approach and demonstrate its validity and power in testing for haplotype interaction association.

**Conclusion:**

Our simulations showed that, for binary trait without covariates, our proposed methods prove to be equal and even more powerful than htr and hapcc which are part of the FAMHAP program. Additionally, our model can be applied to a wider variety of traits and allow adjustment for other covariates. To test the validity, our methods are applied to analyze the association between four unlinked candidate genes and pig meat quality.

## Background

Haplotypes, the linear arrangement of alleles on the same chromosome inherited as a unit, provide a natural framework for testing the association between genetic markers and complex traits more efficiently than separate marker analysis[1]. There is strong evidence that several mutations in *cis *position within a single gene can interact to create a "super allele" that has a large effect on the observed phenotype. The biological explanation for these haplotype effects is that several mutations in a gene cause several amino acid changes in the ultimate protein product, and the joint effect of these amino acid changes can have a much larger influence on the function of the protein product than any single amino acid change. This emphasizes the importance of examining candidate genes by SNP haplotyping. Some studies focus on haplotypes within a given genomic region [2-7]. Because complex traits are presumed to be the results of interaction by a set of genes which may be located in different regions, some methods aim to test gene-gene interaction, and interactions of single markers from different unlinked regions [8-11]. Specifically, Becker *et al*[12] reported a method to deal with haplotype interaction in unlinked regions for a binomial trait. They find the best haplotype combination from the unlinked regions by permutation, which is a modification of Ge *et al*[13]. However, this method could only be applied to case-control association testing, and could not include other covariates.

Generalized Linear Model (GLM) is an extension of the general linear modeling process that allows models to be fitted for several kinds of traits, such as Gaussian, Poisson, Binomial, etc., and allows various covariates. Schaid *et al*[5] introduced score statistics, which are receiving increased attention because they require only computation of the null estimates and are asymptotically equivalent to Wald and likelihood ratio statistics under both null and Pitman alternative hypotheses. Some methods that use score tests based on GLM to test haplotype-trait association have demonstrated the validity and power of this statistic[6]. However, these methods only considered one genomic region. If considering multi-region multi-marker haplotype configurations, a severe multiple-testing problem will occur. To obtain uncorrected *P*-values for a specific marker combination, we use an unnested simulation introduced by Becker and Knapp[2], which is based on the algorithm proposed by Ge *et al*[13].

We propose an alternative approach that uses score statistics based on GLM to build the statistic *T *over which some of the unlinked regions are considered and some markers are chosen at the selected regions. Since the distribution of *T *is generally unknown and is generally not comparable, we replace *T *with *P*^*min *^which is inherited from the algorithm of Becker and Knapp[2]. This simulation method has already been validated by Manly[14] and Hoh *et al*[15], and has systematically been applied to some genetic data[2,12,16,17].

## Simulation study

### Simulation schemes

We conduct a simulation study to evaluate the power and type I error of the association test and to compare our approach with others. The haplotype data are generated in a way similar to that of Roeder *et al*.[18] and Tzeng *et al*[6]. In every simulation scheme, we consider two unlinked regions. We consider that markers are in strong linkage disequilibrium within each region, but markers from different regions are in linkage equilibrium. Therefore, we separately produce two regions by using a modified Hudson's MS program[19]. This program generates data under a coalescent model in which the recombination occurs uniformly over the region. The 4 samples sizes are 50, 100, 200 and 400, respectively. The scaled recombination rate, *ρ *= *N*_*e*_*δ*/*bp*, is set to 4 × 10^-3 ^for the recombination cold spots, and 100 times greater in the hot spots, with the effective population size *N*_*e *_is 1 × 10^4^. The scaled mutation for the entire region, 4*N*_*e*_*μ*/*bp*, is set to be 6 × 10^-4^. Once the haplotypes have been generated, the first step is to restrict the disease or minor allele frequency. In this simulation, we set the allele frequency as 3 levels: 0.1, 0.3, and 0.5. We assume that the middle locus in every gene is the liability locus. Once a liability locus is chosen, a haplotype is defined as a segment of three adjacent SNPs in which the second SNP is the liability locus.

After randomly pairing haplotypes to form individual genotypes, we generate both continuous and binary trait values.

#### Continuous traits

For the Type I error test, we consider two simple models of quantitative traits simulated independently of the liability locus. Let model 1 include only an environmental effect *e*: ***Y ***= ***e***. Let model 2 additionally incorporate a covariate *Z*: ***Y ***= *γ *× ***Z ***+ ***e***. In the models, *e *follows a standard normal distribution with mean 0 and variance 1, and *Z *is generated from a standard normal distribution. For assessing power and the effective selection of the best combination of markers, we also consider two models of quantitative traits simulated in association with the liability locus. Let model 3 decompose the trait value into MRHC effect and environmental effect *e*: *Y *= *g *+ *e*, and model 4 additionally incorporate a covariate *Z*: *Y *= *g *+ *γ *× *Z *+ *e*. In these models, *g *is the sum of all considered genes' effects. For the *i*th gene, *g*_*i *_has a discrete distribution and equals , ,  with probabilities *q*^2^, 2*q*(1-*q*) and (1-*q*)^2^, respectively. As in models 1 and 2, *e *follows a normal distribution with mean *ε *and variance , and *Z *is generated from a standard normal distribution. For simplicity, we set , *ε *= 0, and *γ *= 1. The trait values are generated using the normal penetrance function.  for the first model and  for the second model, where *m *is the number of the genes. We determine  through the heritability *h*^2 ^of all liability loci, which we set at 0.4.

#### Binary traits

We generate binomial phenotypes on the basis of the above continuous traits, and consider four models where the disease prevalence is set to 0.10. If the values of the above continuous traits are more than a given threshold, we set the traits as disease cases, otherwise we set them as control. Let model 5 be the binary trait created from a continuous trait simulated as in model 1, model 6 from model 2, model 7 from model 3, and model 8 from model 4. Binary traits are simulated until an equal number of cases and controls are reached.

The detail of the models can be seen in table 1.

**Table 1:** the 8 models in the trait producing in simulation

**Model name**	**Trait type**	**Factor consider**
Model 1	Continuous traits	include only an environmental effect *e*
Model 2	Continuous traits	include an environmental effect *e *and a covariate
Model 3	Continuous traits	include MRHC effect and environmental effect *e*
Model 4	Continuous traits	include MRHC effect and environmental effect *e *and a covariate
Model 5	Binary Traits	Produce from model 1 above a given threshold
Model 6	Binary Traits	Produce from model 2 above a given threshold
Model 7	Binary Traits	Produce from model 3 above a given threshold
Model 8	Binary Traits	Produce from model 4 above a given threshold

Under all scenarios, we compute the global *P *value with 1500 permutation replicates for each simulated data set. Empirical significance levels and power were computed as the portion of simulated data sets for which the global *P *value was less than or equal to 0.05.

## Results

### Comparison of three models htr, hapcc and HAPGLM

In order to check the validity and the accuracy of our HAPGLM approach, we first carry out simulations under the null hypothesis and compare it with hapcc and htr, which were implemented in the beta version of FAMHAP. htr performs a haplotype trend regression test proposed by Zaykin *et al*[7], and hapcc performs a *χ*^2 ^test for haplotypes proposed by Becker *et al*[12,16]. Here, we use model 5 and 7 to simulate the trait. Haplotypes and trait values are compared according to the frequency (*q *= 0.1, 0.3, 0.5) of the disease allele, and sample size (*n *= 50, 100, 200, 400).

First, we discuss the results under the effect of minor allele frequency for type I error in table 2. Under 12 scenarios, the type I error of the three models is near 0.05, and there are not significant differences between the three models. The results show that our model can approximate to hapcc and htr in accuracy. For the power comparison, table 3 presents worst performance when disease allele frequency is high with small sample sizes, where the power of the global test is not stabilized, especially for the *hapcc*. The reason is that the disease individual prevalence is 0.10, and the percent for the disease is somewhat small, making it difficult to find the significant difference. However, when the sample size is more than 100, the power is near one for the three models. There are no significant differences among hapcc, htr and our method.

**Table 2:** Type I error of three models via 1500 simulations at *α *= 0.05

**Sample Size**	**Minor Allele**	**Model Type**		
		
		***hapcc***	***htr***	***HAPGLM***
50	q = 0.1	0.097(0.079-0.117)	0.048(0.036-0.063)	0.048(0.036-0.063)
	q = 0.3	0.032(0.022-0.045)	0.039(0.028-0.053)	0.044(0.032-0.059)
	q = 0.5	0.029(0.020-0.041)	0.041(0.030-0.055)	0.047(0.042-0.071)
100	q = 0.1	0.036(0.025-0.049)	0.035(0.024-0.048)	0.047(0.035-0.062)
	q = 0.3	0.031(0.021-0.044)	0.072(0.057-0.090)	0.043(0.031-0.057)
	q = 0.5	0.042(0.030-0.056)	0.047(0.035-0.062)	0.051(0.038-0.067)
200	q = 0.1	0.031(0.021-0.044)	0.026(0.017-0.038)	0.055(0.042-0.071)
	q = 0.3	0.051(0.038-0.067)	0.033(0.023-0.046)	0.047(0.035-0.062)
	q = 0.5	0.040(0.029-0.054)	0.067(0.052-0.084)	0.037(0.026-0.051)
400	q = 0.1	0.035(0.024-0.048)	0.037(0.026-0.051)	0.042(0.030-0.056)
	q = 0.3	0.047(0.035-0.062)	0.028(0.019-0.040)	0.041(0.030-0.055)
	q = 0.5	0.028(0.019-0.040)	0.041(0.030-0.055)	0.042(0.030-0.056)

**Table 3:** Power of three models via 1500 simulations at *α *= 0.05

**Sample Size**	**Disease Allele Frequency**	**Model Type**		
		
		***hapcc***	***htr***	***HAPGLM***
50	q = 0.1	0.947	0.947	0.947
	q = 0.3	0.866	0.907	0.968
	q = 0.5	0.666	0.391	0.596
100	q = 0.1	0.977	0.971	0.952
	q = 0.3	0.963	0.927	0.941
	q = 0.5	0.834	0.728	0.917
200	q = 0.1	0.980	0.980	0.968
	q = 0.3	0.981	0.981	0.968
	q = 0.5	0.961	0.912	0.954
400	q = 0.1	0.967	0.981	0.948
	q = 0.3	0.981	0.983	0.974
	q = 0.5	0.934	0.925	0.967

### Three factors analysis for global test

To evaluate the test performance, we describe the results from our power and type I error study that use the above methods with various parameters. Type I error test includes 48 scenarios which include 4 models, 3 minor allele frequencies and 4 sample sizes. As shown in table 4, the type I error stabilizes in all the scenarios. Power test includes 48 scenarios which include 4 models, 3 disease allele frequencies and 4 same sizes. For the power calculations in table 5, the power is adversely affected by the small sample size and high disease allele frequency. Otherwise, if the sample size is at least to 100, the power is preserved. Therefore, we set sample size to test recombination affection and the specific MRHC testing as 100.

**Table 4:** Type I error of global test via 1500 simulations at *α *= 0.05

**Sample Size**	**Minor Allele Frequency**	**Model Type**			
		
		**Model 1**	**Model 2**	**Model 5**	**Model 6**
50	q = 0.1	0.034(0.024-0.047)	0.031(0.021-0.044)	0.048(0.036-0.063)	0.039(0.028-0.053)
	q = 0.3	0.050(0.037-0.065)	0.041(0.030-0.055)	0.044(0.032-0.059)	0.044(0.032-0.059)
	q = 0.5	0.052(0.039-0.068)	0.062(0.048-0.079)	0.047(0.035-0.062)	0.056(0.043-0.072)
100	q = 0.1	0.040(0.029-0.054)	0.032(0.022-0.045)	0.047(0.035-0.062)	0.037(0.026-0.051)
	q = 0.3	0.038(0.027-0.052)	0.044(0.032-0.059)	0.043(0.031-0.059)	0.037(0.026-0.051)
	q = 0.5	0.048(0.036-0.063)	0.044(0.032-0.059)	0.051(0.038-0.067)	0.052(0.039-0.068)
200	q = 0.1	0.045(0.033-0.060)	0.041(0.030-0.055)	0.055(0.042-0.071)	0.031(0.021-0.044)
	q = 0.3	0.048(0.036-0.063)	0.041(0.030-0.055)	0.047(0.035-0.062)	0.042(0.030-0.056)
	q = 0.5	0.047(0.035-0.062)	0.049(0.036-0.064)	0.037(0.026-0.051)	0.041(0.030-0.055)
400	q = 0.1	0.027(0.018-0.039)	0.040(0.029-0.054)	0.042(0.030-0.056)	0.038(0.027-0.052)
	q = 0.3	0.043(0.031-0.059)	0.037(0.026-0.051)	0.041(0.030-0.055)	0.044(0.032-0.059)
	q = 0.5	0.041(0.030-0.055)	0.052(0.039-0.068)	0.042(0.030-0.056)	0.048(0.036-0.063)

**Table 5:** Power of global test via 1500 simulations at *α *= 0.05

**Sample Size**	**Disease Allele Frequency**	**Model Type**			
		
		**Model 3**	**Model 4**	**Model 7**	**Model 8**
50	q = 0.1	0.977	0.824	0.947	0.478
	q = 0.3	0.964	0.920	0.968	0.891
	q = 0.5	0.947	0.625	0.596	0.741
100	q = 0.1	0.934	0.936	0.952	0.937
	q = 0.3	0.979	0.957	0.941	0.889
	q = 0.5	0.965	0.836	0.917	0.887
200	q = 0.1	0.978	0.963	0.968	0.916
	q = 0.3	0.968	0.972	0.968	0.967
	q = 0.5	0.967	0.971	0.954	0.960
400	q = 0.1	0.968	0.967	0.948	0.944
	q = 0.3	0.971	0.947	0.974	0.920
	q = 0.5	0.948	0.958	0.967	0.962

### Recombination analysis for global test

In order to check the effect of recombination on the model, we first consider two different recombination levels at which the diversity of the haplotype is high and low. "High diversity" indicates that a minor or disease locus is located in the region of recombination hot spots and that the number of distinct haplotypes is 6-8; "low diversity" indicates that a minor or disease locus is located in a haplotype block and the number of distinct haplotype is 3-5. In this simulation, we consider three SNPs in each region and we assume equal recombination level. The first recombination level has 2-4 different haplotypes. The haplotype distribution of the second recombination level consists of 6-7 theoretically possible haplotypes. From table 6 and 7, there are some differences between the two diversities on type I error and power, but there are no significant differences between the high and low diversities. That is to say, the proposed method is not significantly affected by the recombination level.

**Table 6:** Type I error of global test for different recombination at *α *= 0.05

**Haplotype Diversity**	**Minor Allele Frequency**	**Model Type**			
		
		**Model 1**	**Model 2**	**Model 5**	**Model 6**
High	0.1	0.046(0.034-0.061)	0.053(0.040-0.069)	0.045(0.033-0.060)	0.043(0.031-0.057)
	0.3	0.051(0.038-0.067)	0.043(0.031-0.057)	0.047(0.035-0.062)	0.038(0.027-0.052)
	0.5	0.058(0.044-0.074)	0.052(0.039-0.068)	0.042(0.030-0.056)	0.042(0.030-0.056)
Low	0.1	0.047(0.035-0.062)	0.041(0.030-0.055)	0.042(0.030-0.056)	0.043(0.031-0.057)
	0.3	0.039(0.028-0.053)	0.044(0.032-0.059)	0.037(0.026-0.051)	0.047(0.035-0.062)
	0.5	0.040(0.029-0.054)	0.051(0.038-0.057)	0.044(0.032-0.059)	0.050(0.037-0.065)

**Table 7:** Power of global for different recombination level at *α *= 0.05

**Haplotype Diversity**	**Disease Allele Frequency**	**Model Type**			
		
		**Model 3**	**Model 4**	**Model 7**	**Model 8**
high	0.1	0.971	0.860	0.955	0.957
	0.3	0.967	0.962	0.961	0.961
	0.5	0.948	0.968	0.953	0.965
low	0.1	0.973	0.977	0.972	0.972
	0.3	0.963	0.944	0.951	0.958
	0.5	0.977	0.933	0.948	0.953

### Analysis for specific MRHC

The score test can easily compute the specific MRHC. In order to study the performance of the proposed method in detecting individual MRHCs, we set 3 disease allele frequencies and 4 model fits. The power for specific MRHC is presented in table 8. It is of interest that the proposed method is robust for detecting the specific MRHC.

**Table 8:** Power for the specific MRHC at *α *= 0.05

**Disease Allele** **Frequency**	**Model Type**			
	
	**Model 3**	**Model 4**	**Model 7**	**Model 8**
0.1	0.981	0.856	0.978	0.946
0.3	0.943	0.962	0.916	0.937
0.5	0.933	0.954	0.946	0.952

### Select the best combination of markers

In order to check the accuracy of our methods to select the best MRHC, the markers of the 2 genes in simulations are 123|456, and the marker 2 and 5 are as the liability loci. The frequency of the disease allele is 0.3. The result shows that for these 2 liability loci combination, the statistic *T *is the largest and the *P *value is the smallest. These are presented in table 9. The combination without these two loci presents low *T *and high *P *value.

**Table 9:** The best ten combinations in two regions with six markers

**Model**	**1**	**2**	**3**	**4**	**5**	**6**	**7**	**8**	**9**	**10**
Model 5	2|5	12|5	23|5	123|5	245	234|5	123|45	12|56	123|56	12|456
Model 6	5	2	2|5	3|5	2|6	23	12	56	2|4	45
Model 7	2|5	12|45	13|456	123|45	2|45	23|45	123|56	12|456	123|45	12|56
Model 8	12|5	2|5	23|5	2|45	12|45	2|56	123|5	23|56	123|45	12|56

The possible number for the combination is 2^6^-1 with 6 markers in 2 regions.

### Application to a pig meat quality dataset

Meat quality is very important in the pig meat production industry. Many candidate genes have been identified that could be used to improve this trait through marker assisted selection (MAS)[20]. The Heart Fatty Acid-Binding (*H-FABP*) gene encodes a type of cytosol protein that transports fatty acids from the cell membrane to other sites where 3-acyl-glyceride and phospholipids are synthesized and fatty acids are oxidized. Gerbens [21-23] discovered *Msp *I, *Hae *II and *Hinf *I polymorphisms of the *H-FABP *gene that is related to intramuscular fat content. Melanocortin-4 Receptor (MC4R) is believed to be a link between feed intake and body weight[24].

Polymorphism of the *MC4R *gene has been reported to be associated with back fat thickness[25]. Adipocyte Determination and Differentiation factor-1 (*ADD1*) can activate or restrain some genes in fat and glucose metabolism. Research has suggested that the *ADD1 *gene can be used as a candidate gene for pork quality[26]. Calpastatin (*CAST*), which is an endogenous inhibitor (Ca^2+ ^dependent cysteine proteinase), plays a central role in the regulation of calpain activity in cellsand is considered to be one of the major modulators of the calpains[27,28]. The *CAST *gene represents an excellent candidate gene for studying variation in pork quality. We aim to find association between multi-region haplotype effects from these candidate regions and meat quality.

Our data set is a sample which includes 93 unrelated fatteners from the following breeds/populations: 18 Meishan, 21 Sutai, 14 Yorkshire × Sutai, 16 Landrace × Sutai and 24 Duroc × Landrace × Yorkshire pigs. 8 polymorphic markers of the preceding genes in the populations have been reported[29,30], which are: 2 in *ADD1*, 1 in *H-FABP*, 1 in *MC4R *and 4 in *CAST*. We code these polymorphic markers as (A1, A2, H1, M1, C1, C2, C3, and C4). The *χ*^2 ^test of these polymorphic markers show that there are significant differences in 5 polymorphic markers (except for A1, A2, C4) between the five populations. We set up single locus models including sex and breed as environmental covariates, for every polymorphic marker, and use statistical software SAS macro GLM for calculations. The results show significant effects at 0.05 level for: A1, H1, C2, C3 and C4 in back fat thickness (BK); A1, C1 and C3 in meat color (MC); A1, H1, C1 and C4 in intramuscular fat content (IMF), and A1 in protein content. To apply our methods, we also incorporate two environmental covariates (sex and breed), and use back fat thickness, tenderness, drip loss, meat color, intramuscular fat content, pH 1 hour after slaughter, pH 24 hours after slaughter, and the content of protein as the dependent variable in the regression model. Table 10 illustrates the marker combination in which raw *P *values are lower at 0.01 level, Compared to single locus model, MRHC analysis can detect more markers which are significantly associated with traits.

**Table 10:** All marker combinations with raw *P *values less than 0.01

**Trait Type**	**Number**	**Marker Combination**
BK^a^	15	3|67, 1|3|568, 1|3|7, 3|58, 2|3|58, 3|568, 3|57, 1|3|5, 1|3|57, 3|567, 1|3|56, 2|3|68, 1|3|78, 2|3|78, 3|578
Tend^b^	34	4|68, 4|56, 578, 568, 4|568, 1|4|58, 1|4|68, 4, 3|56, 1|2|57, 4|567, 3|58, 3|4|5, 1|57, 3|4|58, 3|568, 4|67, 5|67, 4|78, 58, 5678 4|678, 4|58, 4|57, 3|4|7, 3|4|6, 3|578, 1|3|4|5, 1|4|5, 1|3|678, 3|4|578, 1|3|4|8, 1|3|56,1|3|5
DL^c^	4	1|3|678, 3|4|578, 567, 58
MC^d^	10	1|3|4|5, 3|4|678, 1|3|57, 1|568, 1|4|567, 1|4|57, 1|4|578, 1|56. 1|3|56, 1|3|67
IMF^e^	4	1|3|4|5, 1|4|56, 4, 568
pH1^f^	32	1|3|4|5, 1|3|5, 1|3|56, 1|3|57, 1|3|6, 1|3|67, 1|3|7, 1|4|567, 1|4|57, 1|4|578, 1|4|58, 1|4|6, 1|56, 1|578, 3|4|56, 3|4|6, 3|4|678,3|4|7, 3|4|8, 3|56, 3|57, 3|568, 3|57, 3|58, 3|6, 3|67, 3|678, 3|7, 3|78, 4|56, 4|58, 4|6
pH24^h^	5	1|4|8, 3|4|8, 4|6, 58, 8
Protein^i^	8	1|3|6, 5, 1|5, 12|3, 1|4|8, 3|58, 1|3|8, 12|3|4|5

^a ^back fat thickness, ^b ^tenderness, ^c ^drip loss, ^d ^meat color, ^e ^intramuscular fat content, ^f ^PH after 1 hour's slaughter, ^h^PH after 24 hours' slaughter, ^i ^the content of protein. The eight markers (A1, A2, H1, M1, C1, C2, C3, C4) as 1 2 3 4 5 6 7 8, and same as follows.

Additionally, our methods are used to reconstruct the distinct MRHC from the above 4 genes which are in unlinked regions. In table 11, we illustrate the 5 top statistics for MRHCs which consider 8 markers combination.

**Table 11:** The specific MRHC of 4 regions of haplotype interaction

**Trait Type**	**The specfic haplotype configurations in 4 genes**
BK	AG|A|A|AGGA, AG|A|G|GGGA, AG|A|G|AGGA, AG|G|G|AGAA, AG|G|G|AGAA
Tend	AG|A|G|AGGA, AG|A|G|AGGA, AG|A|G|AGGA, GG|A|A|AAAA^a^, GG|G|A|AAAA^a^
DL	AG|A|G|AGAA, AG|A|G|AGGA, AG|G|G|AGAA, GG|G|A|AAAA^a^, AA|A|A|AAAA^a^
MC	AG|A|A|AGGA, AG|A|G|AAGA, AG|A|G|AGAA, AG|A|G|AGGA, AG|G|G|AGAA^a^
IMF	AG|A|G|AGAA, AG|A|G|AGGA, AA|G|G|AAAG^a^, AA|G|A|AAAG^a^, AG|G|G|AAGG^a^
pH1	AG|A|G|AGAA, AG|A|G|AGGA, AG|G|G|AGAA, AG|G|G|AAAA^a^, AG|A|A|AAGA
pH24	AG|A|G|AGAA, AG|A|G|AGGA, AG|G|G|AGAA, AG|A|A|AGGA^a^, AG|A|A|AAGA^a^
Protein	AG|A|G|AGAA, AG|A|G|AGGA, AG|A|A|AGGA, AG|A|A|AGGA^a^, AA|A|A|AAGA^a^

^a ^denote the statistics *z*_*k *_is very low.

## Discussion

Presently many publications have proven that the genetic dissection of complex traits depends not only on the identification of genes involved in disease susceptibility but also on the elucidation of the synergistic role that genes play with other genes and with environmental factors[8-11,31-33]. Therefore, considering unlinked genomic regions simultaneously is desirable. There are two models hapcc and htr in FAMHAP program which can compute the haplotype interaction in unlinked region. For hapcc, Becker *et al*.[12] chose the usual *χ*^2 ^test statistic for contingency tables which can be applied only to case-control traits. For htr, the haplotype trend regression test proposed by Zaykin *et al*[7], chose *F *statistic and could be used for qualitative and quantitative traits, but won't allow other covariates in the model. Our proposed methods are based on score equations for GLMs which allow adjustment of covariates and can model qualitative and quantitative traits. For binary trait without covariate, the type I error and power comparison show that our model has the same power as hapcc and htr, and type I error is as expected. For a small number of markers, the run times for hapcc, htr and HAPGLM are approximately equal. Additionally, our model has obvious advantages. First, our model can be applied to analyze haplotype association across independent regions with adjusting of covariates for a wider variety of traits. Second, the score statistic () of the individual MRHCs can be easily computed.

Our model adopted the simulation method proposed by Becker *et al*.[2,12,17], which can be computationally feasible to deal with the multiple marker combination. For our program, the evaluation of a single simulated data set with 15 markers in 3 regions will take no more than 10 seconds on average on two nodes with 3.0 GHz Intel with 512 MB main memory. In general, it will be possible to simultaneously consider about 600 to 1,200 hypotheses on a standard PC. Our program is very flexible to allow selection of loci and genes for analysis. However, in regions with too many possible haplotype combinations, our program runs out of memory. We need to consider more aggressive trimming parameters or other haplotype estimation algorithms. For example, we will improve our program by using the haplotype ancestral cluster idea to cluster rare haplotypes with similar ancestral haplotypes, which was used by Tzeng *et al*.[34]. Haplotypes of the entire block can be represented by a smaller set of SNPs which are referred to as tag SNPs[35]. In order to analyze more markers, it will be helpful to select tag markers at each region and to carry out the analysis on the set of these markers. Tag SNPs selection will save run-time, and we plan our further research along this path.

In this research, we proposed markers from different regions which are proposed to be in linkage equilibrium. Markers from different regions can be in linkage disequilibrium, but the methods can allow such markers as if they were in the same region.

Since the current model assumes that the subjects are independent of each other (i.e., unrelated), it is critical to extend the current approach to account for the correlations between subjects, given their family data. Therefore, further studies are needed to address the impact and modeling strategies with regard to the assumptions in the model. This model is restricted to outcomes that can be placed in the generalized linear model framework. An extension to failure-time data could also be placed in the framework.

## Conclusion

It is quite common that the genetic architecture of complex traits involves many genes and their interactions. Therefore, dealing with multiple unlinked genomic regions simultaneously is desirable. We developed a regression-based approach which can be applied to a wider variety of traits and allow adjustment for other covariates to assess the interactions of haplotypes that belong to different unlinked regions. Multiple marker combinations at each unlinked region are also considered. In addition, HAPGLM can be downloaded for free at: , user: ylhu0323, password: public.

## Methods

### Contribution to multi-region haplotype configurations

Consider *R *unlinked genomic regions, and *m*_*r *_observed markers for each of *n *unrelated individuals in region *r*. Further, we suppose that markers within each region are in strong linkage disequilibrium, while markers from different regions are in linkage equilibrium. What we wish to investigate is whether some of the genomic regions are associated with the phenotype of interest via some of the markers from each region.

Let *G*^*r *^be the multi-locus genotype of an individual at region *r*, and *h*^*r *^be a haplotype of region *r*. If the haplotypes  and  are compatible with *G*^*r*^,  is then called a haplotype explanation of *G*^*r*^. Obviously, for a given *G*^*r*^, there may be several haplotype explanations.

First, we use the expectation-maximization (EM) algorithm[5] to obtain the maximum-likelihood estimates of the haplotype frequencies at each of the unlinked regions. After we pooled the rare haplotypes (with estimated frequencies <0.001) into a single group, we adopt the following formula ([12] to compute the likelihood weights of all haplotype explanations of each region for each individual,

(1)

where *G*^*r *^is the multilocus genotype of a fixed individual at region *r*. Let  be the set of unordered haplotype explanation, which are compatible with *G*^*r*^. let  be the estimated frequency of haplotype , the sum in the denominator runs over all possible haplotype explanations, and the Kronecker symbol *δ *is defined as *δ*_*j*, *k *_= 1 if *j *= *k*, and *δ*_*j*, *k *_= 0 if *j *≠ *k*, where *j *and *k *is the pair of haplotypes at region *r*. The "~*r*" is all the pair haplotypes in region *r *for each individual.

Let *G *= (*G*^1^, *G*^2^, and, *G*^*R*^) be the multi-region genotype of an individual and  be a possible multi-region haplotype explanations (MRHEs) that are compatible with (*G*^1^, *G*^2^, and, *G*^*R*^). Let  denote a multi-region haplotype configuration (MRHC). An MRHE is formed by two MRHCs from the two gametes, but there may be 2^R ^MRHCs for a given MRHE, some of which may be the same. We construct an *n *× *h *matrix with rows referring to the *n *individuals and columns referring to the different MRHCs. Cell *x*_*ef *_of this matrix denotes the contribution of individual *e *to MRHC *f*, and can be calculated according to the following equation,

(2)

### Regression model with MRHC

Let *y *denote an *n *× *1 *vector of measured phenotypes of a trait, *α *denote an *h *× *1 *vector of the effects for the MRHCs, *β *denote the regression parameters for the intercept and environmental factors,  be the contribution matrix obtained above, and  denote the design matrix corresponding to measured environmental factors. Then we have the following generalized linear model (GLM):

(3)

Let *Z *= *X*_*e*_|*X*_*g *_and *γ *= (*α*|*β*). Then, the likelihood of trait *y*_*i *_for subject *i*, given the vector *Z*_*i*_, can be expressed as a GLM for exponential family data[36] according to

(4)

where *a*, *b*, and *c *are known functions, and *ϕ *is the dispersion parameter. To implement the score statistics for different types of traits, we need only assume a distribution for the trait and to make the appropriate substitutions for the expected value of the trait, , the dispersion parameter *a*(*ϕ*), the ratio *b*"(*η*)/*a*(*ϕ*) and the link function. (see table 1 of [5]).

### Score test for incorporating contribution of MRHC

We derive score statistics to test the null hypothesis of no association between MRHC and trait, *H*_0 _: *α *= 0. Let *ζ *denote the vector of nuisance parameters (*β*, *μ*, *ϕ*). The likelihood function for (*α*, *ζ*) on the basis of the data  according to Tzeng *et al*[6] is

(5)

Where *P*(*x*_*g*, *i*_) is the contribution of individual *i *to MRHCs.

The score function for *α *is the partial derivative of the likelihood equation (5), with respect to *α*. The resulting score statistic, denoted by *S*_*α*_, is the score function evaluated at the restricted maximum-likelihood estimate under the null hypothesis. *S*_*α *_is the statistic that we use to test MRHC effect; in appendix A, we show the following result:

(6)

where  and  are the restricted maximum-likelihood estimated under the null hypothesis, and *E*(*X*_*g*, *i*_|*G*_*i*_) is the contribution of individual *i *to MRHC under the observed multi-region genotypes, *G*.

To test the association between MRHC and trait that adjusts for other covariates, we need to compute the variance of *S*_*α *_under the null hypothesis *H*_0_: *α *= *0*. Under *H*_0_, *S*_*α *_is asymptotically distributed as multivariate normal[36]. We consider the generalized score test, which would ensure the asymptotic null *χ*^2 ^distribution even under model misspecification[5]. Define *θ *= (*α*, *ζ*) and let *V*_*α *_denote the variance of *S*_*α*_, the equation can be expressed according to Louis[37] and Tzeng *et al*[6] as:

(7)

where,





In appendix B, we show the above result.

With the above results, we can compute a global score statistic according to

(8)

The score statistic is distributed asymptotically as *χ*^2 ^with degrees of freedom equal to the rank of *V*_*α*_.

Schaid *et al*[5] proved that the score function for *α *and the score function for haplotype probabilities are independent under the null hypothesis, so that the covariance between the two score functions is zero. Since the contribution to each MRHC is estimated from the haplotype frequency that is used to calculate the score statistic *S*_*α*_, the variance of the score statistic is not penalized by the use of estimated haplotype frequencies.

In this framework, we can readily compute score statistics for each MRHC according to[5]:

(9)

where *z*_*k *_follows *χ*^2^(1) under the null hypothesis *H*_0_: *α *= 0.

The *P *value  is assessed via simulation. In each replicate of this simulation, a sample is constructed in which the sample trait and environmental covariate of each individual are randomly permuted at the same time, and the score test statistic is computed again. Let  denote the value of the test statistic obtained for the *i*th replicate. Then  is the fraction of permutation replicates resulting in a test statistic greater than or equal to the test statistic of the real data, i.e., , with *t *denoting the number of permutation replicates and || denoting the number of elements of .

### Testing more than one hypothesis

If we select *m *markers in several genes, there would be 2^m^-1 marker combinations. To test 2^m^-1 combinations with associated raw *P *values, and declare the global *P *value the significance level for our analysis would lead to another multiple-testing problem. In order to avoid nested simulation, we use the method which Becker and Knapp[2] adapted from Ge *et al*[13]. The basic idea is that, to test B = 2^m^-1 marker combinations, global *P *is estimated by the proportion of permutation samples with min _*B*_P_*t *_smaller than that in the observed data, where *t *is the simulation time. For each marker combination *B *∈ *B *and for each permutation replicate *i *= 1,..., *t*, the raw *P *value of the *i*th permutation replicate is calculated as

(10)

For *i *> 0,  is the minimum of the uncorrected *P *values over all MRHC in the *i*th permutation replicate. So the *P *value for the global hypothesis *H*_0 _is calculated as:

(11)

This permutation method is explained in more detail in[2].

## Authors' contributions

YH developed the statistical model, carried out the software implementation, and made the simulation design and drafted the manuscript. JS helped with discussion both in theoretical developments and English copyediting. YP helped with discussion in theoretical developments, as well as in drafting the manuscript. QW, XZ and HZ contributed with discussion on theoretical aspects and drafting the manuscript. CL and LS contributed with the experimental data. All authors read and approved the manuscript.

## Appendix A

Let *S*_*α *_(*Y*, *G*, *X*_*e*_, *α*, *ζ*) denote the score function of the data (*Y*, *G*, *X*_*e*_) for *α*. As set forth by[37], *S*_*α *_(*Y*, *G*, *X*_*e*_, *α*, *ζ*) is the expectation of the complete-data score function given the observed data--that is,



## Appendix B

For the expected Fisher information function of the observed data (*Y, G, Xe*), *I *is



where



The hybrid estimate of *I *is obtained by replacing the nonzero entries of *I *with the observed Fisher information (denoted by *i*):



Hence, equation (7) can be simplified as



Recall that



and that[37] proposed



so that


